# Assessment of Different Root Canal Preparation Techniques with Rotary Nickel-Titanium Instruments by Novice Students

**DOI:** 10.3390/dj6030046

**Published:** 2018-09-04

**Authors:** Andreas Bartols, David W. Christofzik, Matthias Krummel, Christian Friedrichs, Tim Pousset, Birte Größner-Schreiber, Christof E. Dörfer

**Affiliations:** 1 Dental Academy for Continuing Professional Development, 76135 Karlsruhe, Germany; 2 School for Dental Medicine, Christian-Albrechts-University Kiel, Clinic for Conservative Dentistry and Periodontology, 24105 Kiel, Germany; David.Christofzik@uksh.de (D.W.C.); MKrummel@tonline.de (M.K.); praxis@endo-endo.de (C.F.); Birte.Groessner-Schreiber@uksh.de (B.G.-S.); Christof.Doerfer@uksh.de (C.E.D.); 3 Dentist in Private Practice, 24159 Kiel, Germany; 4 Endodontic Specialist in Private Practice, 24118 Kiel, Germany; 5 Dentist in Private Practice, 24107 Kiel, Germany; timpousset@googlemail.com

**Keywords:** assessment, crown-down, endodontics, endodontic techniques, FlexMaster, Mtwo, Reciproc, single-file, single-length

## Abstract

This study investigated which preparation strategy for root canals leads to the best technical preparation quality, and moreover, which is perceived to be performed best by novice students. Sixty-four students were recruited to prepare one simulated root canal with each of the following: FlexMaster files (F), Mtwo files (M), and Reciproc files (R). After preparation, the students assessed the different instrument systems through a questionnaire. The technical quality of the root canal preparations was evaluated by the centering ratio of the preparation. A total of 186 prepared root canals were submitted for evaluation. With R, significantly better centered preparations were achieved when compared to M and F (*p* < 0.001). The students evaluated R faster than M and F, and evaluated F significantly (*p* < 0.05) slower than R and M. M was rated as the easiest system to learn and to handle, as well as the best at reaching the working length; therefore, it was evaluated as the overall favorite of the students. A difference was found between the students’ perceptions and their achieved technical quality of root canal preparations.

## 1. Introduction

The overall technical preparation quality with nickel-titanium (NiTi) rotary instruments is generally better rated than root canal preparation with hand instruments [1]. Modern NiTi instruments for rotary root canal preparation have been shown to be more reliable in terms of preparation faults, the straightening of root canals, and instrument fractures [2].

Hand instrumentation is taught at universities worldwide as the standard basic technique for root canal preparation. It is a safe technique with regard to instrument fracture, even for inexperienced operators [3]. The integration of NiTi rotary preparation into undergraduate education curricula at universities has not been very widespread [4], but studies have shown that even novice operators can achieve better root canal preparation quality with NiTi rotary instruments than with hand instruments [3,5]. One of the most commonly used rotary NiTi systems in student education in Germany is the FlexMaster system (F) (VDW, Munich, Germany) for crown-down preparations [6]. More recent instruments such as single-length files, e.g., Mtwo (M) (VDW, Munich, Germany), or single-file systems, e.g., Reciproc (R) (VDW, Munich, Germany), have rarely been introduced into student education. While all of these instruments have shown overall good shaping quality in root canals [7,8], little is known about the performance and assessment of these different preparation techniques by inexperienced operators who have had no previous contact with such different root canal preparation strategies.

Therefore, we sought to investigate how students without any experience in rotary root canal preparation would perform using these different instrumentation systems. In addition, we subjectively assessed the three different root canal preparation strategies: crown-down preparation represented by F instruments, single-length preparation represented by M, and single-file reciprocating preparation represented by R (all instruments VDW, Munich, Germany).

## 2. Materials and Methods 

As the present study did not interfere with the psychological or physical integrity of the students; the participation was completely voluntarily; the analyses of data were performed completely anonymously and the questionnaires did not contain any identifying information; and no patients were involved in this study, according to the Professional Code for Physicians of the Medical Council of the State of Schleswig-Holstein and according to the Institutional Review Board (IRB), an approval for this study was not necessary. All students were informed about the character of the study; therefore, further informed consent was not necessary according to the local IRB.

### 2.1. Operators

For our study, we recruited 64 students without any experience in rotary root canal preparation. All had experience with manual root canal preparation with stainless steel instruments. In a cross-over design, they were randomly assigned to one of three study groups. In every group, the students used the root canal instruments F, M, and R in a different order (FMR, MRF, or RFM) to minimize bias due to learning effects from previous preparation techniques. All students were introduced to rotary root canal preparations generally—and to each of the three different systems specifically—through standardized lectures. Each student prepared one simulated root canal per instrumentation system to practice, and immediately following prepared another canal that was included in the study for evaluation. After that, they moved to the next instrumentation system and prepared one block to practice and one for evaluation.

### 2.2. Simulated Root Canals

It has been a common observation of ours and other researchers that the shape of simulated root canals in resin blocks differs due to production-related fluctuations. Therefore, we developed a software tool that made it possible to evaluate each simulated root canal under the same conditions and with the same precision.

**Figure 1:**
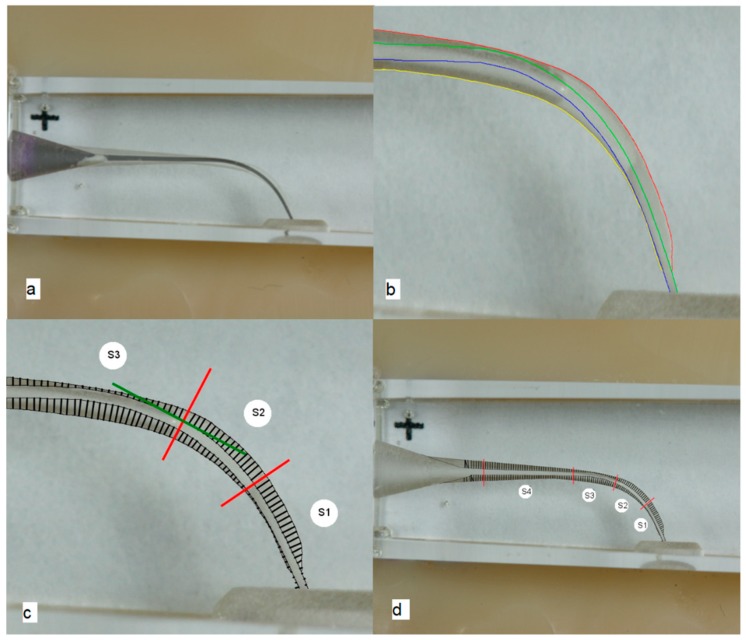
(**a**) Superimposed images of the unprepared and prepared canal. (**b**) Traced canal outlines. (**c**) Placement of tangents and measurement points placed. (**d**) Sections and measurement points placed over the complete canal.

A total of 192 resin training blocks (VDW, Munich, Germany) with simulated root canals with a curvature angle of 40° were used for this study. All canals were stained with toluidine blue to produce a better contrast for the upcoming image processing. Then, the blocks were fixed in an exactly fitting plastic template (Luxatemp, DMG, Hamburg, Germany) that was mounted with a LEGO plate (LEGO A/S, Billund, Denmark) to a table tripod (Repro-Copy Outfit PF-4, Nikon, Düsseldorf, Germany). A digital standardized photograph was taken using a remote-controlled camera (Canon EOS D400, Krefeld, Germany) with a macro objective (Canon EF 100 mm/1:2.8 USM, Krefeld, Germany). After the root canal preparation, another standardized image was taken under the same conditions and with the same equipment. Next, the pictures were superimposed in Photoshop CS5.1 (Adobe Systems, San José, CA, USA) (Figure 1a). In both images, the outlines of the root canals were traced with a blue, green, red, and yellow colored line (as pure RGB colors) (Figure 1b). Afterwards, it was possible to perform an automated evaluation of the images with a software tool that was specially developed by our research group. In the first step, the program placed tangents on the outer surfaces of the prepared root canals. In the second step, perpendicular lines to these tangents were constructed at the intersection with the outer root canal surface (Figure 1c). The program measured the difference between the unprepared and the prepared root canal surfaces. Moreover, the inner and the outer preparation spaces were related to each other (Figure 1d). In an ideally centered root canal preparation, the relation of both spaces would be exactly 1.0. The centering ratio was calculated not only for the complete canal length, but also for four sections of the root canal with S1 at the apex and S4 in the coronal part of the root canal. The three apical sections included 20 measurement points, and the coronal section 40 measurement points (Figure 1d).

In addition, unintended events such as via falsa and preparations beyond the working length (WL) as well as instrument fractures were counted.

### 2.3. Root Canal Instrumentation

The WL of the simulated root canals was measured with an ISO 10 K-file (VDW, Munich, Germany) as the total canal length minus 0.5 mm. Next, the resin blocks were masked with opaque tape (Durapore, 3M, Neuss, Germany). Before mechanical instrumentation, a glide-path was prepared up to ISO 15 with stainless steel K-files (VDW, Munich, Germany). The students used a VDW.SILVER RECIPROC motor (VDW, Munich, Germany) with the predefined settings for each rotary or reciprocating file. For each resin block, new hand and rotary/reciprocating files were used. The F instruments were used in rotary motion with the crown-down approach in the sequence: Introfile, .06/25, .06/20, .04/30, .04/25, .02/25, .02/30, .04/30 (VDW, Munich, Germany). The M instruments were used in rotary motion with the single-length approach with the sequence: .04/10, .05/15, .06/20, .06/25, .05/30 (VDW, Munich, Germany). The R25 (VDW, Munich, Germany) was used in reciprocating motion as one single-file. After each instrument had been used, the canal was rinsed with 2.0 mL of water using a plastic syringe with a 27 gauge needle (Sterican 0.40 × 25 mm, B. Braun, Melsungen, Germany). Patency was maintained with an ISO 15 K-file (VDW, Munich, Germany). Detailed information regarding the materials and instruments used in this study, including the rotation and torque values for the different root canal instruments, can be found in the Supplementary Materials (S1).

### 2.4. Study Questionnaires

After the preparation of the simulated root canals, the students were instructed to subjectively assess all three root canal preparation systems with a standardized questionnaire (see Supplementary Materials, S2). The students were asked to rate the systems regarding “preparation speed”, “ease of understanding the instrument sequence”, “ease of learning the system”, “ease in handling”, and “ease in reaching the working length” using a five-stage ordinal scale with the ranks 1 = very slow/very difficult, 2 = slow/difficult, 3 = indifferent, 4 = fast/easy, 5 = very fast/very easy. Finally, the students were asked which of the three systems they assessed was their overall favorite.

### 2.5. Statistical Analysis

The statistical analysis was performed using GraphPadPrism 6 (GraphPad Software, Inc., La Jolla, CA, USA). Analysis of variance (ANOVA) and post hoc Tukey HSD (Honestly Significant Difference). tests were used to compare the canal centering ratio between the different groups and to analyze the ordinal scale ratings of the questionnaire. Analyses of the different unintended events were performed using the χ^2^ test.

## 3. Results

Of the 64 students, 62 submitted 186 prepared resin blocks with simulated root canals that could be included in the statistical analyses. In each experimental group (R, M, and F), every student prepared one simulated root canal, resulting in 62 prepared resin blocks per group. Two students stated at the time of root canal preparation that they already had experience with rotary instrumentation. Therefore, their preparations and evaluations were excluded from the study.

In the overall analysis of root canal straightening, R maintained the original root canal geometry significantly better (*p* < 0.001) compared to the other systems, with a mean centering ratio of 0.58 (SD (Standard Deviation) 0.09), followed by F and M with centering ratios of 0.50 (SD 0.08) and 0.49 (SD 0.12), respectively (Table 1). The Tukey multiple comparisons showed that R was significantly better centered than F (*p* < 0.001) and M (*p* < 0.001). There were no significant differences between F and M (*p* = 0.72).

The analysis of the apical part of the root canal (S1) showed that R maintained the original root canal geometry better than the other systems with a mean centering ratio of 0.29 (SD 0.24), followed by M with a centering ratio of 0.24 (SD 0.24) and F with 0.18 (SD 0.18). The differences were statistically significant (*p* = 0.026). The Tukey multiple comparisons showed that R was significantly better centered than F (*p* = 0.019), but the differences were not statistically significant between R and M (*p* = 0.474) and between F and M (*p* = 0.27).

The analysis of the intermediate part of the root canal (S2) showed that R maintained the original root canal geometry better than the other systems with a mean centering ratio of 0.43 (SD 0.22), followed by M with 0.38 (SD 0.24) and F with 0.29 (SD 0.11). The differences were significant (*p* = 0.001). The Tukey multiple comparisons showed that R was significantly better centered than F (*p* < 0.001). Additionally, M was significantly better centered than F (*p* = 0.033). There were no significant differences between R and M (*p* = 0.35).

The analysis of S3 showed that F maintained the original root canal geometry better than the other systems with a mean centering ratio of 0.57 (SD 0.06), followed by R with 0.49 (SD 0.09) and M with 0.44 (SD 0.10). The differences were significant (*p* < 0.001). The Tukey multiple comparisons showed that F was significantly better centered than M (*p* < 0.001) and R (*p* = 0.001), while R was significantly better centered than M (*p* = 0.01).

The analysis of the most coronal part of the canal (S4) showed that R maintained the original root canal geometry better than the other systems with a mean centering ratio of 0.49 (SD 0.12), followed by F with 0.38 (SD 0.13) and M with 0.34 (SD 0.14). The differences were significant (*p* < 0.001). The Tukey multiple comparisons showed that R was significantly better centered than F (*p* < 0.001) and M (*p* < 0.001). There were no differences between F and M (*p* = 0.38).

**Figure 2:**
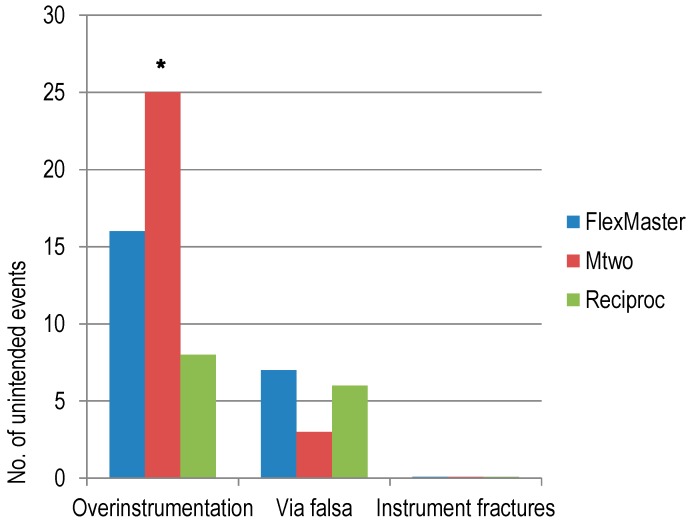
Unintended events during root canal preparation with the different strategies: crown-down (F), single-length (M), and single-file (R) (χ^2^, *N* = 62 per group, * indicates significant differences with *p* < 0.01 between all groups).

Fifteen (8.1%) preparations resulted in a via falsa. Two (3.2%) via falsas occurred with M, six (9.6%) with R, and seven (11.3%) with F instruments (Figure 2). There were no significant differences (χ^2^; *p* = 0.218). In 45 (24.2%) cases, preparations beyond the WL occurred during root canal preparation. With R, there were seven (11.3%) preparations beyond the WL, 15 (24.2%) with F, and 23 (37.1%) with M. There were significant differences between all systems (χ^2^; *p* = 0.004). File fractures did not occur in any of the experimental groups.

**Figure 3:**
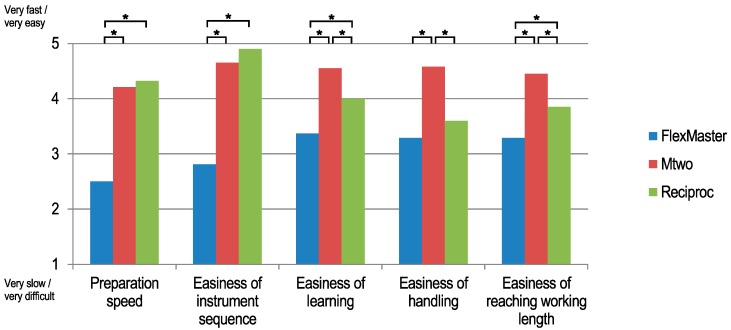
The students’ subjective questionnaire evaluation of the three root canal preparation strategies: crown-down (F), single-length (M) and single-file (R) (ANOVA with post hoc Tukey HSD; * indicates significant differences with *p* < 0.05 between groups).

The return rate of the questionnaire was 96.8% (62 of 64). In general, F was rated as the slowest and most difficult system. F was significantly slower or more difficult (*p* < 0.05) compared to at least one of the other systems in every item asked in the questionnaire (see Table 2 and Figure 3). The students evaluated R as the fastest preparation system, whereas F was rated as significantly slower compared to both M and R (*p* < 0.001). R and M were rated as having significantly easier instrument sequences than F (R and M vs. F *p* < 0.001; R vs. M *p* = 0.054). M was rated as the easiest system to learn (M vs. F and R *p* < 0.002), the easiest system to handle (M vs. F and R *p* < 0.001), and the system with which the working length could be more easily reached when compared to the other systems (M vs. F and R; *p* < 0.004). Overall, 59.7% of the students rated M as their favorite root canal preparation system, with 34.4% favoring R and 4.8% favoring F. The differences were statistically significant (χ^2^; *p* < 0.001).

## 4. Discussion

It is generally accepted that rotary root canal instrumentation leads to better preparation geometries than hand instrumentation, even with unexperienced operators such as novice students [3,9,10,11,12]. Differences between the rotary preparation quality performed by experienced and inexperienced operators are sometimes small [9,13].

Novice operators without any experience in rotary root canal preparations were able to prepare simulated root canals safely regarding instrument fractures, as no fractures occurred with any of the systems tested. The students rated M as their favorite root canal preparation system overall. Regarding centering ability, R instruments were better than the other systems and therefore resulted in better preparation geometries. Preparation faults such as via falsas and preparations beyond the WL occurred at a higher frequency than normal with all systems.

To the knowledge of the authors, no previous studies have investigated the quality of root canal preparation as well as students’ assessment of different root canal preparation concepts. A goal in modern endodontic education is to teach concepts for root canal preparation that achieve optimal results regarding preparation quality and can be easily understood and implemented by novice dental students.

A study that compared experienced and unexperienced operators regarding the preparation quality of simulated root canals with single-file reciprocating WaveOne instruments concluded that, regardless of the operators’ level of experience, the canal preparations all were of very good quality [13]. We also found this in our study in which the students achieved better technical preparations with the R single-file reciprocating system compared to the other systems. This applied to both the overall centering ratio as well as the ratio at the crucial apical sections of S1 and S2, and the coronal section, S4. Only in section S3 was F better centered. A recent study found that M and R instruments maintained the original curvature in natural teeth without significant differences [7]. The finding that R instruments were better centered than the other instruments at the apical part of the canal can be attributed to the fact that instrumentation systems with a sequence of different instruments have a prolonged preparation time, and thus have more opportunity to straighten the apical part of the root canal to a greater extent. Additionally, R25, as a single-file, has a smaller diameter at the tip than the other multiple-file systems. Moreover, the softer material of the training resin blocks in contrast to dentine [14] contributed to more pronounced canal transportation in multiple-file systems like M and F.

In this study, we investigated not only the technical quality of root canal preparations, but also which of the three preparation strategies (crown-down, single-length, or single-file) were subjectively preferred by the novice students. To our knowledge, no previous studies have compared all three methods in such a setting. To date, comparisons between hand and rotary preparations of root canals have mainly been employed [3,10,12,15], with better results achieved by the rotary preparation. In our study, there was no comparison with hand preparation, but the older crown-down method with F instruments was less-centered as well as more laboriously rated by the novice students compared to the other methods.

It has been described in the literature that student reception of rotary root canal preparations is very positive in contrast to hand preparations [10,15,16]. A comparison between different rotary systems has not been available thus far. In this study, the students subjectively evaluated the crown-down method with F instruments regarding preparation speed, understandability of instrument sequence, ease of learning, ease of handling, and ease in reaching the working length as the least favorable system compared with M and R. R and M were subjectively rated as faster than F. This is consistent with other studies that have objectively measured the preparation times of the different systems [7,8]. R, as a single-file system, was consequently rated as the system with the easiest instrument sequence. However, M was rated as having the best overall handling, being the easiest to learn, being the easiest to reach the working length with, and was the overall favorite for nearly 60% of the students. This was in contrast to the significantly better centered preparations with R as well as the significantly lower number of preparations beyond the WL. However, since the students rated the different systems subjectively and without knowing the quality of their own preparations, we do not know whether the survey results would have been different with additional knowledge of the preparation quality.

In this study, no instrument fractured. This may be explained by the single-use nature of the instruments. In other studies involving students, fracture rates were reported to be between 1.3% and 5.56% [3,10]. Preparation faults, such as via falsas and preparations beyond the WL, were frequent in this study, which was in accordance with another study performed with inexperienced operators [11].

The use of simulated root canals in resin blocks has been widely described [9,17] and is a valid method for comparing different root canal instruments. The advantages of resin blocks include the standardization of the canals to a certain degree and the elimination of tooth-related factors, such as different dentine properties, different anatomies, etc. [13]. Even if, so far, the canal geometries have been assumed to be equal between the blocks, commercially available simulated root canals lack standardization [18]. To eliminate the occurrence of measurement faults resulting from production-related deviations in the root canal shapes, we developed a software tool that was able to partially automate the measurements. Therefore, it was possible to generate a large number of measurements along the root canal that could be used to calculate the centering ratios of different sections and of the whole root canal. With our software tool, it was possible to compare the prepared root canals regardless of certain aberrations. However, the preparation of natural root canals may be more complicated than that of models due to the properties of natural dentine [13,17].

## 5. Conclusions

Novice students achieved better centering ratios for the preparation of simulated root canals with R compared to M and F instruments, especially in the crucial apical sections of the root canal. The M system was the overall favorite for the students, especially regarding handling, ease of learning, and reaching the working length. Further evaluations are needed to uncover the reasons for the differences between the students’ perceptions and the actual achieved quality of root canal preparations.

## Figures and Tables

**Table 1:** Mean centering ratios and SDs of the different systems at different canal sections (S1: apical, S4: coronal) and of the complete canal (ratio of 1.0 = centered).

Instrument	*N*	Centering Ratio									
		S1		S2		S3		S4		Complete Canal	
		Mean	SD	Mean	SD	Mean	SD	Mean	SD	Mean	SD
Flexmaster	62	0.18 ^a^	0.18	0.29 ^a^	0.11	0.57 ^a^	0.06	0.38 ^a^	0.13	0.51 ^a^	0.08
Mtwo	62	0.24 ^a,b^	0.24	0.38 ^b^	0.24	0.44 ^b^	0.10	0.34 ^a^	0.14	0.49 ^a^	0.12
Reciproc	62	0.29 ^b^	0.24	0.43 ^b^	0.22	0.49 ^c^	0.09	0.49 ^b^	0.12	0.58 ^b^	0.09

^a–c^: Different superscript letters in the same column indicate statistically different mean values between groups at *p* < 0.05 (ANOVA with post hoc Tukey HSD).

**Table 2:** Mean values of students’ subjective questionnaire evaluation of the three root canal preparations strategies (rank 1 = very slow/very difficult to rank 5 = very fast/very easy).

Instrument	*N*	Question									
		How Fast Do You Rate the Preparation Speed?		How Easy Is It to Understand the Instrument Sequence?		How Easy Is It to Learn the System?		How Easy Is the Handling of the System?		How Easy Is It to Reach the Working Length?	
		Mean	SD	Mean	SD	Mean	SD	Mean	SD	Mean	SD
Flexmaster	62	2.50 ^a^	0.95	2.81 ^a^	0.90	3.37 ^a^	0.98	3.29 ^a^	1.01	3.29 ^a^	1.17
Mtwo	62	4.21 ^b^	0.58	4.65 ^b^	0.48	4.55 ^b^	0.59	4.58 ^b^	0.53	4.45 ^b^	0.80
Reciproc	62	4.32 ^b^	0.90	4.90 ^b^	0.30	4.00 ^c^	1.04	3.60 ^a^	1.02	3.85 ^c^	1.05

^a–c^: Different superscript letters in the same column indicate statistically different mean values between groups at *p* < 0.05 (ANOVA with post hoc Tukey HSD).

## Supplementary Materials

The following are available online at http://www.mdpi.com/2304-6767/6/3/46/s1, Table S1: Detailed information of the instruments and materials used in this study, Questionnaire S2: Study questionnaire for the students’ evaluation of the different root canal preparation systems.
