# Supplementary material for: Assessment of Different Root Canal Preparation Techniques with Rotary Nickel-Titanium Instruments by Novice Students
Source: Dent J (Basel). 2018 Sep 4;6(3):46. doi: 10.3390/dj6030046 (PMC6162720; doi:10.3390/dj6030046)
Supplement: Supplementary file 1 [file dentistry-06-00046-s001.zip › S2 questionnaire mechanical root canal preparation.docx]

| **Questionnaire for mechanical root canal preparation** | | |
| --- | --- | --- |
| Place No: | Code of resin block: | Date: |

Please rate the different mechanical root canal preparation systems (FlexMaster, Mtwo and Reciproc) with respect to the different criteria in the corresponding columns with the values 1 to 5 (1 = very slow / very difficult, 2 = slow / difficult, 3 = indifferent, 4 = fast / easy, 5 = very fast / very easy)

| **System**  Criteria | **FlexMaster** | **Mtwo** | **Reciproc** |
| --- | --- | --- | --- |
| How fast do you rate the preparation speed? |  |  |  |
| How easy is it to understand the instrument-sequence? |  |  |  |
| How easy is it to learn the system? |  |  |  |
| How easy is the handling of the system? |  |  |  |
| How easy is it to reach working length? |  |  |  |

Which system is your overall favourite for root canal preparation?

⃝ FlexMaster

⃝ Mtwo

⃝ Reciproc
